# Adolescents’ mental health and maladaptive behaviors before the Covid-19 pandemic and 1-year after: analysis of trajectories over time and associated factors

**DOI:** 10.1186/s13034-022-00474-x

**Published:** 2022-06-10

**Authors:** Laura Pedrini, Serena Meloni, Mariangela Lanfredi, Clarissa Ferrari, Andrea Geviti, Annamaria Cattaneo, Roberta Rossi

**Affiliations:** 1 Unit of Psychiatry, IRCCS Istituto Centro San Giovanni Di Dio Fatebenefratelli, Via Pilastroni, 4, Brescia, Italy; 2 Statistics Service, IRCCS Istituto Centro San Giovanni Di Dio Fatebenefratelli, Via Pilastroni, 4, Brescia, Italy; 3 Biological Psychiatry, IRCCS Istituto Centro San Giovanni Di Dio Fatebenefratelli, Via Pilastroni, 4, Brescia, Italy; 4 Department of Pharmacological and Biomolecular Sciences, University of Milan, Milan, Italy

**Keywords:** Adolescence, Mental health, Covid-19, Maladaptive behaviors, Stress, Depression, Emotional regulation, Anxiety, Risk factors, Longitudinal

## Abstract

**Background:**

Adolescents have been deeply exposed to negative consequences of social distancing imposed by Covid-19. There is a lack of longitudinal studies regarding the impact on adolescents of this unfavorable condition, and their results are controversial. The aim of the present prospective study is to assess psychopathological symptoms in adolescent students over time and to evaluate what type of impact the Covid-19 pandemic had on adolescents. Moreover, the association between mental health indexes, potential risk and resilience factors is explored.

**Methods:**

Psychopathological symptoms (i.e., anxiety, depression, stress, emotional dysregulation, maladaptive behaviours), and potential risk and resilience factors (i.e., childhood trauma, emotional regulation skills, family function, personality traits) were assessed among a sample of 153 students (72% female; mean age 16.1 ± 0.49), living in a medium-size city in the north of Italy, at two time points: before the outbreak of the Covid-19 pandemic (November 2019–January 2020) and 1 year later (April–May 2021).

**Results:**

After 1 year, we found an increase in mean scores on anxiety, stress for future uncertainty, and higher frequency of maladaptive behaviours. By contrast, the level of stress related to social domains (i.e., school attendance, romantic relationships, peer pressure) decreased. Dysfunctional emotional regulation skills, childhood trauma, low family functioning, and specific personality traits were associated to higher psychopathological symptoms. Cluster analysis detected three groups of youths based on their change over time in psychopathological symptoms: those who worsened (N = 23; 15%), improved (N = 55; 34%), or remained stable (N = 75; 46%). After controlling for baseline mental health status, those adolescents reporting increase in self-harm (OR = 2.61; p < 0.001), binge-drinking (OR = 3.0; p = 0.007), aggressiveness (OR 1.92; p = 0.004), and binge-eating (OR 2.55; p = 0.003) were more likely to present a worsened mental health condition.

**Conclusion:**

The present results suggest that the Covid-19 pandemic seems to have had a different impact on subgroups of students. Indeed, we found a global worsening of psychological well-being only in a subgroup of adolescents, otherwise other students remained stable or improved. Increased frequency of maladaptive behaviors was found as a predictor of worsened mental health, therefore interventions to strengthen emotional regulation strategies are warranted. Finally, the decrease of stress in social domains could be due to reduction of potential triggering situations, thus indicating only a temporary beneficial effect that requires careful monitoring.

**Supplementary Information:**

The online version contains supplementary material available at 10.1186/s13034-022-00474-x.

## Background

Adolescents were adversely affected by the Covid-19 pandemic. Indeed, restrictive measures aimed to reduce the spread of the virus, such as social distancing and quarantine, caused an interruption of adolescents’ daily routine, whom experienced a prolonged period of physical isolation from their peers, community networks, and extended family. This is relevant as social relationships and interactions are strictly related to identity development, that is a crucial stage and task during adolescence [1]. Moreover, in a period of heightened emotional reactivity, restrictions to engage in sport activities may have contributed to increased difficulties in emotional regulation. Finally, fear for personal and family members’ health represented another relevant source of stress. Indeed, vaccination campaigns started in January 2021, but for adolescents the vaccine was accessibile only later on in the summer, as in most European countries [2].

Several cross-sectional studies had been conducted on adolescents’ mental health during the initial phase of the pandemic, and results consistently showed high rates of depressive and anxiety symptoms [3, 4]. However, because of the observational nature of the studies, it was not possible to infer any conclusions about the exact impact of Covid-19 on mental health in the young population. A recent review underlined that only few longitudinal studies had provided data over time on adolescents [5]. Longitudinal studies showed contrasting results with some reporting no changes [6], some reporting worsened mental health conditions [7–9], and others reporting some improvements after the pandemic [10]. It has been argued that contrasting results could be due to context-related factors, such as the geographical areas where the studies took place [5] or the timing of the assessment [9]. Moreover, it has been recommended to analyze which psychological factors modulate the reactions to the pandemic [11]. Indeed, adolescents’ well-being during pandemic was found to be closely related to specific variables such as mental health before the pandemic [10], emotional regulation skills [8], and family functioning [10, 12].

Furthermore, other two relevant factors that could be considered as predictors of mental health status during the Covid-19 pandemic are childhood trauma and personality traits. Adverse childhood experiences are associated with higher risk for mental health problems and reduced emotion regulation ability [13, 14]. Moreover, there is evidence that specific personality traits may constitute vulnerability factors predisposing to the development of psychopathological disorders or engaging in maladaptive behaviors [15–17]. Since both of these constructs are intrinsically related to the concepts of oneself and others, as well as to expectancies about the future, they can have a direct influence on the way people react to difficult situations [18].

Studies exploring the factors involved in the different trajectories of mental health condition are needed in order to identify those adolescents that are more vulnerable to the negative consequences of the Covid-19 crisis, and to suggest key targets for interventions. The present study has three aims. The first aim is to prospectively evaluate depressive symptoms, anxiety symptoms, perceived stress, emotional dysregulation, and the occurrence of maladaptive behaviors in a sample of adolescent students before the Covid-19 pandemic and one year after. Secondly, to assess whether the Covid-19 pandemic had different impacts on adolescents’ mental health. Our hypothesis is that the Covid-19 pandemic influenced mental health in different ways: youths could show a worsening of their mental health status, or an improvement, or a stable condition. Finally, to explore the association between potential risk or resilience factors and the pattern of change on mental health over time.

## Methods

### Participants

This is a prospective observational study as part of a randomized clinical trial (RCT) on psychoeducational interventions targeting emotional dysregulation in adolescents (ClinicalTrials.gov ID: NCT04349709). The sample of the present paper is a convenience and self-selected sample made up of 8 classes belonging to three high-schools located in Brescia, a city in Northern Italy. The classes followed different academic courses: 2 from a humanities path, 2 from a scientific path and 2 from a languages path. Specifically, in the months preceding the outbreak of the Covid-19 pandemic (November 2019–January 2020) 161 students attending grade 11 (Junior year) were enrolled and underwent a baseline assessment (T0). After the state of emergency was declared, all the activities part of the project had to be suspended, therefore the students did not receive any psychoeducational intervention or further assessments. One year later, during April and May 2021, the same sample of participants was asked to fill out the questionnaire for the second time (T1). A total of 153 students completed both the assessments at T0 and T1 which were included in the analysis of the present study.

The RCT was approved by the local Ethics Committee (approval 40/2019 and 13/2021) and was carried out following the ethical standards presented in the 1964 Declaration of Helsinki. After a detailed and extensive description of the study, written informed consent was obtained both at T0 and T1 from all subjects.

### Assessments

Sociodemographic data (age, gender, parents’ educational level and employment, family structure) was collected. In addition, at T1 students were asked to report their experience during the Covid-19 pandemic. Specifically, they were asked to report if they had contracted the Covid-19 virus, if one their family members had contracted Covid, if they were admitted to hospital for Covid-19, or if any of their family members had died from Covid-19.

The following assessment tests were completed at T0 and T1:

#### Patient health questionnaire-9 (PHQ-9)

The PHQ-9 is a 9-item self-report questionnaire measuring depression severity. The sum of all the items results in the total score. Different levels of severity of depression can be distinguished according to a variety of cut-off scores [19]. For the purpose of this study, we used a PHQ-9 total score ≥ 10 as the cut-off to identify major depression (sensitivity of 88% and specificity of 88%) [19]. PHQ-9 has demonstrated excellent internal validity (Cronbach’s alpha coefficient higher than 0.85) and test–retest reliability [19].

#### Screen for child anxiety related emotional disorders (SCARED)

The SCARED is a 38-item self-report questionnaire measuring symptoms of anxiety. Specifically, it includes different subscales measuring symptoms of panic disorder, generalized anxiety disorder, separation anxiety disorder, school anxiety, and social anxiety. High scores indicate higher anxiety symptoms. The SCARED showed good internal consistency (Cronbach’s alpha coefficient from 0.74 to 0.93 for the total score and each of the five factors) and test–retest reliability [20, 21].

#### Adolescent stress questionnaire (ASQ)

The ASQ asks respondents to indicate how stressful the situations described in each item had been for them during the past year. The ASQ questionnaire includes the following domains: home life, school performance, school attendance, romantic relationships, peer pressure, teacher interaction, future uncertainty, school-leisure conflict, financial pressure. The Cronbach’s alpha for the complete ASQ was 0.93, and item-total correlations ranged from 0.33 to 0.70 [22].

#### Difficulties in emotion regulation scale (DERS)

The DERS is a 36-item self-report questionnaire measuring emotion regulation. The sum of all the items results in the total score. Moreover, this scale includes six subscales: non-acceptance of emotions, difficulties engaging in goal-directed behaviors, impulsivity, lack of emotional awareness, limited emotion regulation strategies, lack of emotional clarity. The DERS exhibits good reliability (Cronbach’s alpha coefficients ranged from 0.76 to 0.89 for the subscales) [23].

#### Checklist for impulsive behaviors (CIB)

This CIB is a checklist specifically designed for this project to measure the frequency of maladaptive behaviors. Participants are asked to indicate on a 5-point Likert scale the frequency of maladaptive behaviors in the past month (1 = never; 2 = only one time; 3 = once a week; 4 = two or three times a week; 5 = at least 4 time a week). The change in the frequency of each behavior was coded as a T0-T1 difference on ordinal 5-points Likert scale.

The following questionnaires were filled out only at T0:

#### Childhood trauma questionnaire (CTQ)

The CTQ is a 28-item self-report questionnaire that measures early adverse experiences such as emotional, physical, and sexual abuse, emotional and physical neglect. The questionnaire is widely used and it showed good psychometric properties with Cronbach’s alpha coefficient = 0.97 for the total score [24]. For the purpose of this study, we used the total score.

#### Family assessment device (FAD)

The FAD is a 36-item self-report questionnaire measuring a family’s ability to work together to satisfy the basic needs of its members. For the purpose of this study, we used the total score. Statistical analysis showed that FAD has an adequate internal consistency with Cronbach’s alpha = 0.94 [25].

#### DBT-ways of coping checklist (DBT-WCCL)

The DBT-WCCL is a 59-item self-report questionnaire measuring functional skills (Skills Subscale) and dysfunctional skills (Dysfunctional Coping Subscale). The scale showed good internal consistency (Cronbach’s alpha coefficients for the subscales ranged from 0.84 to 0.96) and content validity [26].

#### Personality inventory for DSM-5 (PID-5)

The PID-5 is a 220-item self-report questionnaire measuring 25 personality traits, and 5 higher-order trait domains. For our analyses, we exclusively selected five higher-order trait domains: negative affect, detachment, antagonism, disinhibition, and psychoticism. The scale showed good internal consistency (Cronbach’s alpha coefficients greater than 0.90 for all 5 higher-order trait domains [27].

### Statistics

Descriptive statistics (frequencies and percentages for categorical variables, means and standard deviations for continuous variables) were performed. The level of socio-economic status (SES) was calculated by considering the level of education and employment of both parents. Specifically, we divided level of education in 3 categories (primary + middle school = 1, high school diploma = 2, post-secondary degree = 3) and employment in 2 categories (unemployed/housewife + unqualified = 1, qualified + business = 2); then we calculated the SES score related to each student by summing level of education and employment scores for each parent, and then summing the parent scores. The level of SES ranges from 4 to 10 for two-parent families. Specifically, it can be categorized in the following categories: low (SES score ≤ 6); medium (SES score equal 7 and 8); high (SES score ≥ 9). For single-parent families the SES score ranges from 2 to 5 and for computational reasons was multiplied by two.

Gaussianity of variables’ distribution was investigated for the adoption of proper tests and models. T-tests and Chi-squared tests were performed to assess the association of mental health indexes and maladaptive behaviours with gender and SES. For the longitudinal evaluation of mean change of Gaussian distributed variables, paired t-tests were performed, while linear mixed models were performed where it was necessary to correct for other variables. McNemar tests were used to compare changes of percentage distribution across time. Correlation analysis between mental health indexes and risk factors was performed by using Spearman’s rho correlation index.

The longitudinal changes of the four mental health indexes were split into three categories: improved, stable and worsened on the basis of the range of each index. Specifically, for each index, students fell into the following categories: stable category if the change (T0–T1) of the index was around zero ± 10% of its range (defined as maximum-minimum value); improved category if the change was larger than zero + 10% of its range, and worsened category if the change was lower than zero − 10% of its range (see footnote Table 4).

The Multiple Correspondence Analysis (MCA) was performed in order to study the association between the 3 categories (improved, stable or worsened) of each index in terms of geometric distance. The outcome of this analysis was represented in a unique two-dimensional space plot showing the relationships among categories. Categories that are placed in the same quadrant, or that are close enough, suggest an association [28]. A subsequent K-means cluster analysis was carried out to detect subject stratifications (clusters) in terms of change in mental health indexes. K-means method is a hypothesis-driven approach that reallocates subjects into a number (set a priori) of groups, based on each subject’s distance from the cluster means’ vector (centroid). Each subject is assigned to the cluster with the nearest centroid [29]. K-means method was applied to the changes (T0–T1) of the four mental health indexes in order to identify homogeneous clusters of subjects in terms of these changes. The idea behind the application, in turn, of MAC and K-means is that: if associations between categorical variables emerge through the MCA, it is legitimate to expect a clustering of subjects who belong to the associated categories.

Finally, the association between clusters of subjects, found with K-means method, and maladaptive behaviors and other clinical scales, was assessed by using Spearman’s rho correlation analysis and univariate multinomial logistic regression models (clusters of subjects as dependent categorical variables). The association assessed by multinomial logistic regressions was quantified by the odds-ratios (OR). All regressions were adjusted for the status of mental health at baseline, gender, SES and Covid-19 experience. The latter variables were defined as follows: (i) experience of hospitalization of a family member and/or (ii) experience of death of a family member from Covid-19. The variable status of mental health at baseline was defined through the hierarchical cluster analysis (with Ward’s method) on mental health indexes at baseline, in order to detect homogeneous groups of subjects in terms of mental health status (see Additional file 1). In addition, Chi-squared tests were used to assess associations between clusters of subjects with gender, SES and Covid-19 experience.

All tests were two-tailed, and the probability of a type I error was set at *p* < 0.05. The analyses were performed by using software R [30], with packages *FactoMineR* and *cluster* for the MCA and clustering methods respectively; and by using IBM SPSS Statistics for Windows, version 26 (IBM Corp., Armonk, N.Y., USA) for descriptive statistics and multinomial logistic models.

## Results

### Characteristics of the sample

Table 1 shows sociodemographic and clinical characteristics of the sample (N = 153). Participants were mainly female (72%) and the average age was of 16 years old. The SES was substantially equally distributed into three categories: n = 37 (25.9%) participants with low SES, n = 55 (38.4%) with medium SES, and n = 51 (35.7%) with high SES. The level of SES was not associated to clinical indexes, nor with maladaptive behaviors (all p > 0.05).

**Table 1:** Sociodemographic and clinical characteristics of the sample at baseline

	N (%) or mean (SD)
Age	16.09 (0.49)
Gender	
Male	43 (28.3%)
Female	109 (71.7%)
Family structure	
Traditional	120 (84.3%)
Monoparental/adoptive/reconstituted	23 (15.1%)
Mother’s level of education	
Primary school	16 (11%)
Middle school	20 (13.7%)
High school	55 (37.7%)
Post-secondary school	55 (37.7%)
Father’s level of education	
Primary school	22 (14.9%)
Middle school	39 (26.4%)
High school	48 (32.4%)
Post-secondary school	39 (26.4%)
Mother’s employement	
Unemployed or housewife	33 (21.8%)
Businesswoman	37 (24.5%)
Qualified professional	74 (49%)
Unqualified professional	7 (4.6%)
Father’s employement	
Unemployed	3 (2%)
Businessman	39 (26%)
Qualified professional	104 (69.3%)
Unqualified professional	4 (2.7%)
CTQ Total	31.59 (6.56)
FAD Total	113.28 (15.05)
DBT-WCCL Functional Skills	1.58 (0.41)
DBT-WCCL Dysfunctional Coping	1.32 (0.64)
PID-5 negative affect	1.39 (0.60)
PID-5 detachment	0.98 (0.44)
PID-5 antagonism	0.73 (0.48)
PID-5 disinhibition	1.05 (0.44)
PID-5 psychoticism	0.78 (0.48)

*CTQ* childhood trauma questionnaire; *FAD* family assessment device; *DBTWCC* DBT-ways of coping checklist; *PID-5* personality inventory for DSM-5

About half of the students (N = 70; 46%) reported that at least one of their family members contracted Covid-19. A total of 24 (16%) students reported that at least one of their family members had been hospitalized for Covid-19 treatment, and N = 15 (10%) students reported that at least one of their family members died from Covid-19. One out of ten (N = 19; 12%) students contracted Covid-19.

### Mental health indexes: comparison over time

Table 2 shows the mean score comparison of mental health indexes measured at T0 and T1.

**Table 2:** Mean scores on mental health indexes over time

	T0 Mean (SD)	T1 Mean (SD)	P-value^#^
PHQ-9 Total (range 0–27)	7.8 (4.7)	8.3 (4.8)	0.261
SCARED Total (range 38–114)	65.3 (12.1)	67 (12.6)	0.002
Panic disorder	20.2 (5.8)	20.4 (5.6)	0.383
Generalized anxiety	19.4 (3.9)	20.4 (4.2)	0.001
Separation anxiety	11.2 (2.5)	11.1 (2.6)	0.574
School anxiety	6.8 (1.9)	7.6 (2.3)	< 0.001
Social anxiety	7.6 (2.4)	7.7 (2.6)	0.400
ASQ Total (range 27–135)	76 (18.7)	69.5 (16.1)	< 0.001
Home life	11.9 (4.2)	10.2 (4.1)	< 0.001
School performance	10 (3.2)	10.2 (3.4)	0.501
School attendance	6.7 (2.1)	5.6 (2.1)	< 0.001
Romantic relationships	6.4 (3.5)	4.5 (3)	< 0.001
Peer pressure	8.9 (4.6)	6.1 (2.8)	< 0.001
Teacher interaction	7.5 (4.1)	7.8 (3.6)	0.419
Future uncertainty	9.6 (3.5)	10.5 (3.7)	0.002
School leisure conflict	10.6 (3.2)	11.2 (3.2)	0.072
Financial pressure	4.4 (2.6)	3.4 (2)	< 0.001
DERS Total (range 36–180)	87.9 (22.8)	90.1 (21.2)	0.212
Non-acceptance	12.8 (5.3)	12.9 (5.2)	0.893
(Difficulties engaging in) goal-directed behaviour	15.6 (4.7)	15.8 (4.4)	0.711
(Lack of) emotional awareness	15.9 (4.6)	16.8 (5.1)	0.031
Impulsivity	12.7 (4.7)	13.2 (4.4)	0.138
(Limited) strategies	18.1 (7.2)	19 (6.8)	0.102
(Lack of) clarity	12.9 (4.2)	12.6 (4.2)	0.455

*PHQ* patient health questionnaire-9; *SCARED* screen for child anxiety related emotional disorders; *ASQ* adolescent stress questionnaire; *DERS* difficulties in emotion regulation scale

^#^P-value of paired t-test

There was an increase of anxiety symptoms as detected by the SCARED total mean score (p = 0.002), generalized anxiety (p = 0.001) and school anxiety (p < 0.001) subscales. By contrast, no differences were found on panic symptoms, separation anxiety, and social anxiety subscales over time. According to the ASQ’s results, there was an overall reduction of the amount of perceived stress during last year. More in detail, there was a reduction in perceived stress for the following domains: home life (p < 0.001), school attendance (p < 0.001), romantic relationships (p < 0.001), peer pressure (p < 0.001), and financial pressure (p < 0.001). By contrast, there was a significant increase in the perceived stress for future uncertainty (p = 0.002).

Regarding depressive symptoms, there was not a significant difference in PHQ-9’s total mean score over time. Similarly, there was not a difference in the proportion of students with a PHQ-9 total score above the cut-off score at T0 and T1 (respectively, N = 53; 35% and N = 56; 37%) (p = 0.791). Considering youths with a PHQ-9-total score above cut-off at T0 (N = 53), half of them (N = 26; 49%) still showed a score above cut-off at T1, and the remaining (n = 27; 51%) showed score below cut-off at T1. Considering those with PHQ-9-total score below cut-off at T0 (N = 100), one third of them (N = 30; 30%) showed a score above cut-off at T1, and the remaining (N = 70; 70%) still showed score below cut-off at T1.

There was no significant difference in DERS’s total mean score over time, nor for most of the DERS subscales. Indeed, the lack of emotional awareness subscale significantly increased from T0 to T1 (p = 0.031).

### Maladaptive behaviours: comparison over time

Table 3 shows the frequency of maladaptive behaviors in the last month at T0 and T1. Results show a significant increase in the proportion of students reporting unprotected sex (p = 0.027), self-harm ideation (p = 0.005), self-harm behaviors (p = 0.021), binge eating episodes (p = 0.001), and aggressive behaviors (p = 0.003). More in detail, compared to T0 the increase of students reporting maladaptive behaviors ranges from  + 8% for unprotected sex, self-harm behaviors and binge-eating,  + 13% for self-harm ideation, up to  + 15% for aggressive behaviors. By contrast, there is no difference in the proportion of students reporting binge-drinking and cannabis use.

**Table 3:** Frequency of maladaptive behaviours over time

	Never	Only 1 time	About once a week	At least 2 or 3 times a week	P-value^§^
Binge drinking					
T0	130 (85%)	18 (11.8%)	5 (3.3%)	0 (0%)	0.166
T1	136 (88.9%)	11 (7.2%)	5 (3.3%)	1 (0.7%)	
Cannabis					
T0	142 (92.8%)	7 (4.6%)	3 (2%)	1 (0.7%)	0.484
T1	134 (87.6%)	10 (6.5%)	6 (3.9%)	3 (2%)	
Unprotected sex					
T0	148 (96.7%)	1 (0.7%)	1 (0.7%)	3 (2%)	0.027
T1	136 (88.9%)	8 (5.2%)	3 (2%)	6 (4%)	
Self-harm ideation					
T0	140 (91.5%)	10 (6.5%)	2 (1.3%)	1 (0.7%)	0.005
T1	120 (78.4%)	16 (10.4%)	11 (7.2%)	6 (3.9%)	
Self-harm behaviors					
T0	140 (96.7%)	5 (3.3%)	0	0	0.021
T1	136 (88.9%)	7 (4.6%)	7 (4.6%)	3 (1.9%)	
Binge eating					
T0	139 (90.8%)	10 (6.5%)	3 (2%)	1 (0.7%)	0.001
T1	127 (83%)	10 (6.5%)	8 (5.2%)	8 (5.2%)	
Aggressiveness					
T0	95 (62.1%)	38 (24.8%)	11 (7.2%)	9 (5.9%)	0.003
T1	72 (47%)	39 (25.5%)	23 (15%)	19 (12%)	

^§^P-value of McNemar test

### Category of changes over time in mental health indexes

**Fig. 1:**
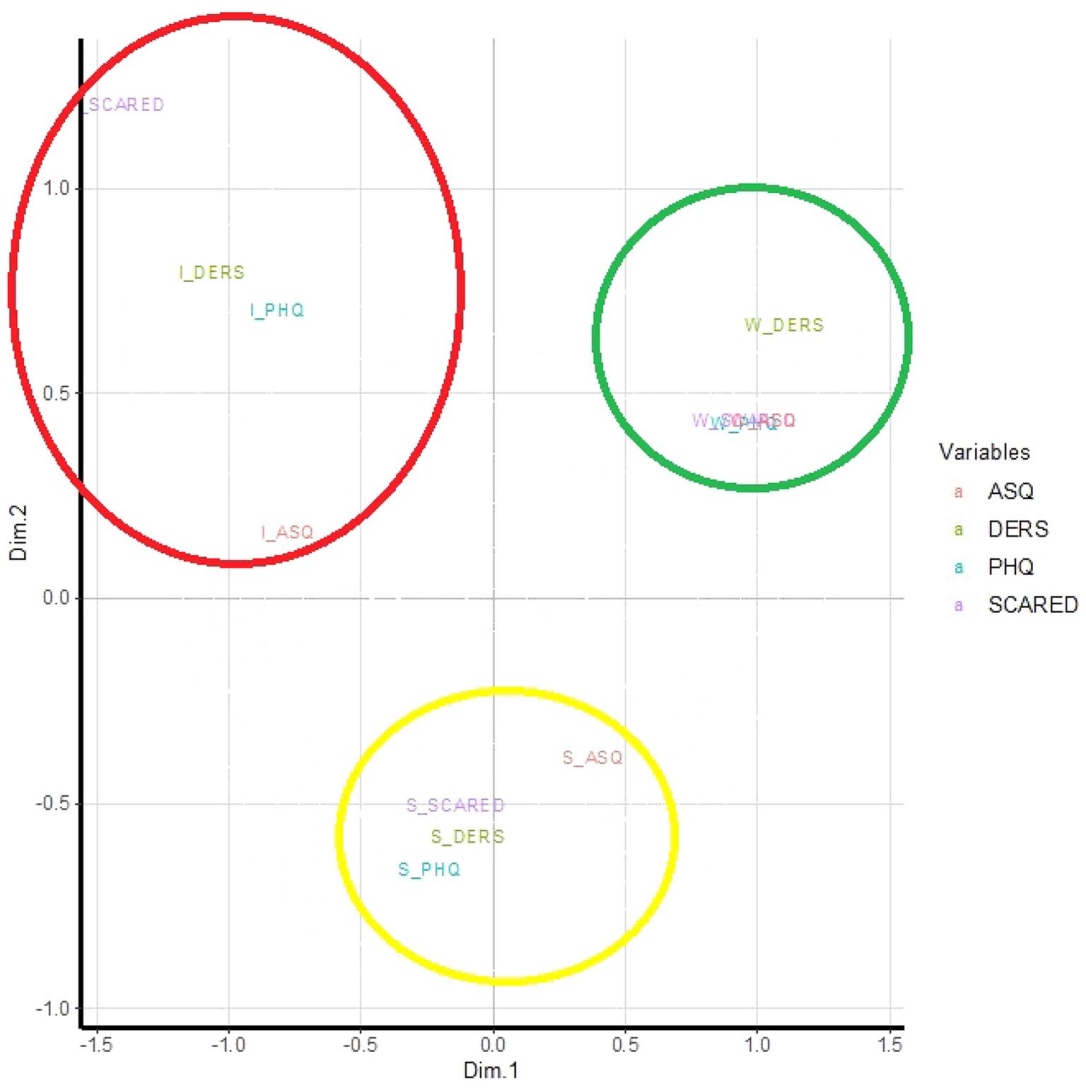
MCA biplot showing association between the categories of change over time in mental health indexes. Categories of scales: I_DERS: improved DERS, S_DERS: stable DERS, W_DERS: worsened DERS; I_PHQ-9: improved PHQ-9, S_PHQ-9: stable PHQ-9, W_PHQ-9: worsened PHQ-9; I_SCARED: improved SCARED, S_SCARED: stable SCARED, W_SCARED: worsened SCARED; I_ASQ: improved ASQ, S_ASQ: stable ASQ, W_ASQ: worsened ASQ

Figure 1 shows the results of MCA performed on the categories of change (i.e., worsened, stable, improved) of PHQ-9, DERS, SCARED and ASQ. As the table shows, the categories of change were highly interrelated across measures: the improvement of one of the variables was strictly associated to the improvement of the others. Indeed, in Fig. 1, the improved categories of the four mental health indexes are close to each other. The same trend was found for the worsening pattern, as well as the stable pattern. Notably, the improved and worsened categories appeared well separated along the horizontal first dimension, whereas the stable categories were separated from the other categories along the second vertical dimension: this suggests substantial different profiles of the students as they show improvement, stability and worsening in a consistent way on all mental health indexes.

**Fig. 2:**
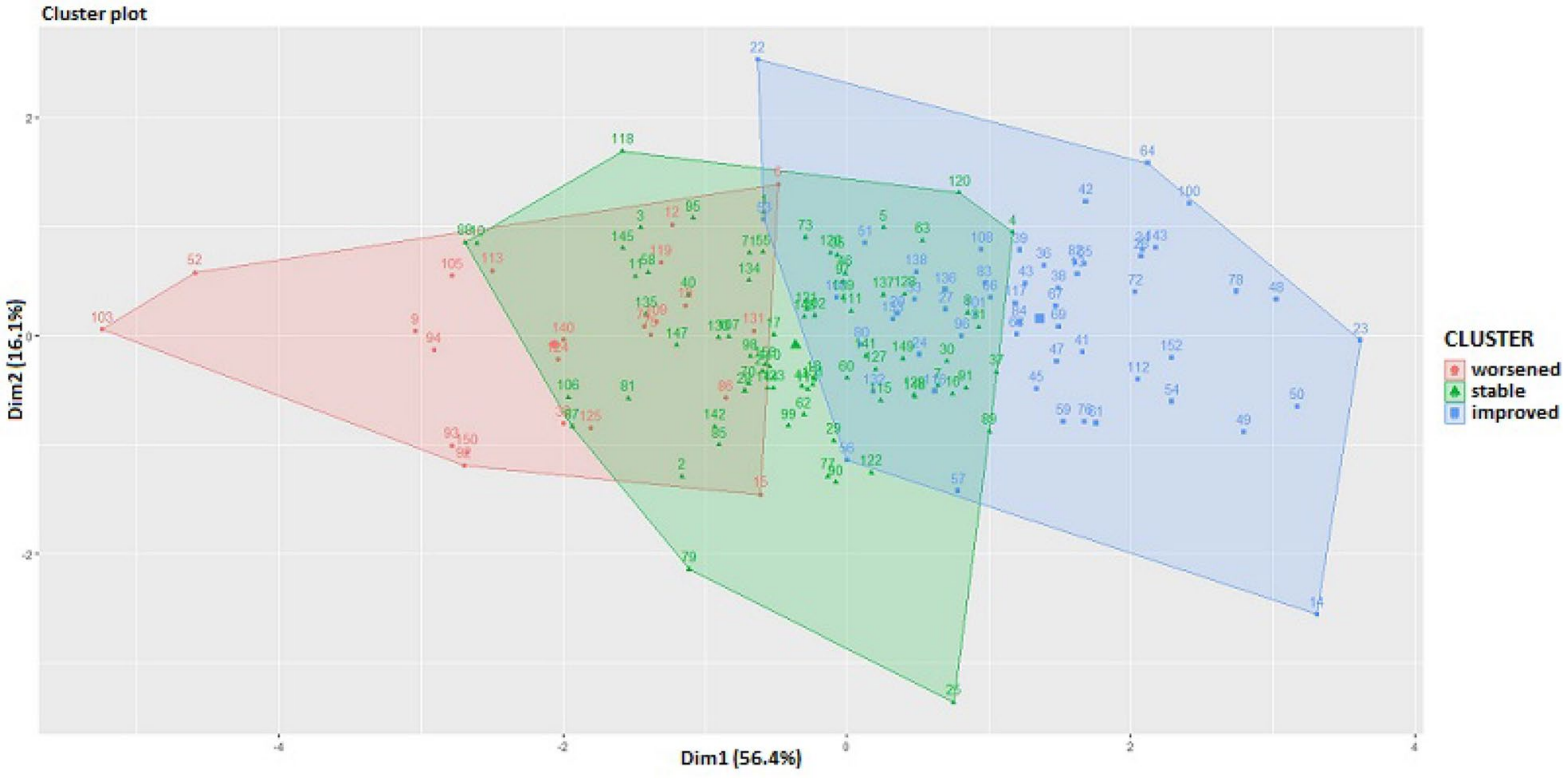
K-means cluster analysis (k = 3) performed on the changes change in mental health indexes

The coherence of changes across all the four indexes (i.e., depression, anxiety, stress, emotional dysregulation) found through MCA, lead us to hypothesize the presence of homogeneous groups of students in terms of change (computed as differences T0–T1) of these indexes. To detect these groups, a k-means cluster analysis (with number of cluster set equal to k = 3) was performed. Figure 2 shows the results outlining that three groups of participants were identified: those who worsened over time on all the questionnaires (N = 23, red cluster), those who improved over time on all the questionnaires (N = 55, blue cluster), and those who remained stable over time on all the questionnaires (N = 75, green cluster). In particular, the worsened cluster appeared quite far from the other two clusters: the group of worsened students reported an average increase of 38 points on the DERS, 4 points on the PHQ, 10 points on the SCARED, 8 points on the ASQ (see Additional file 1: Table S1), highlighting substantial differences between this cluster and the other two.

The categories of change in mental health were not associated with gender (p = 0.072), nor with SES (p = 0.972). There was no significant association between category of change in mental health status over time and having experienced hospitalization or bereavement of family member from Covid-19 (p-value = 0.750).

### Associations between mental health indexes and risk or resilience factors

**Fig. 3:**
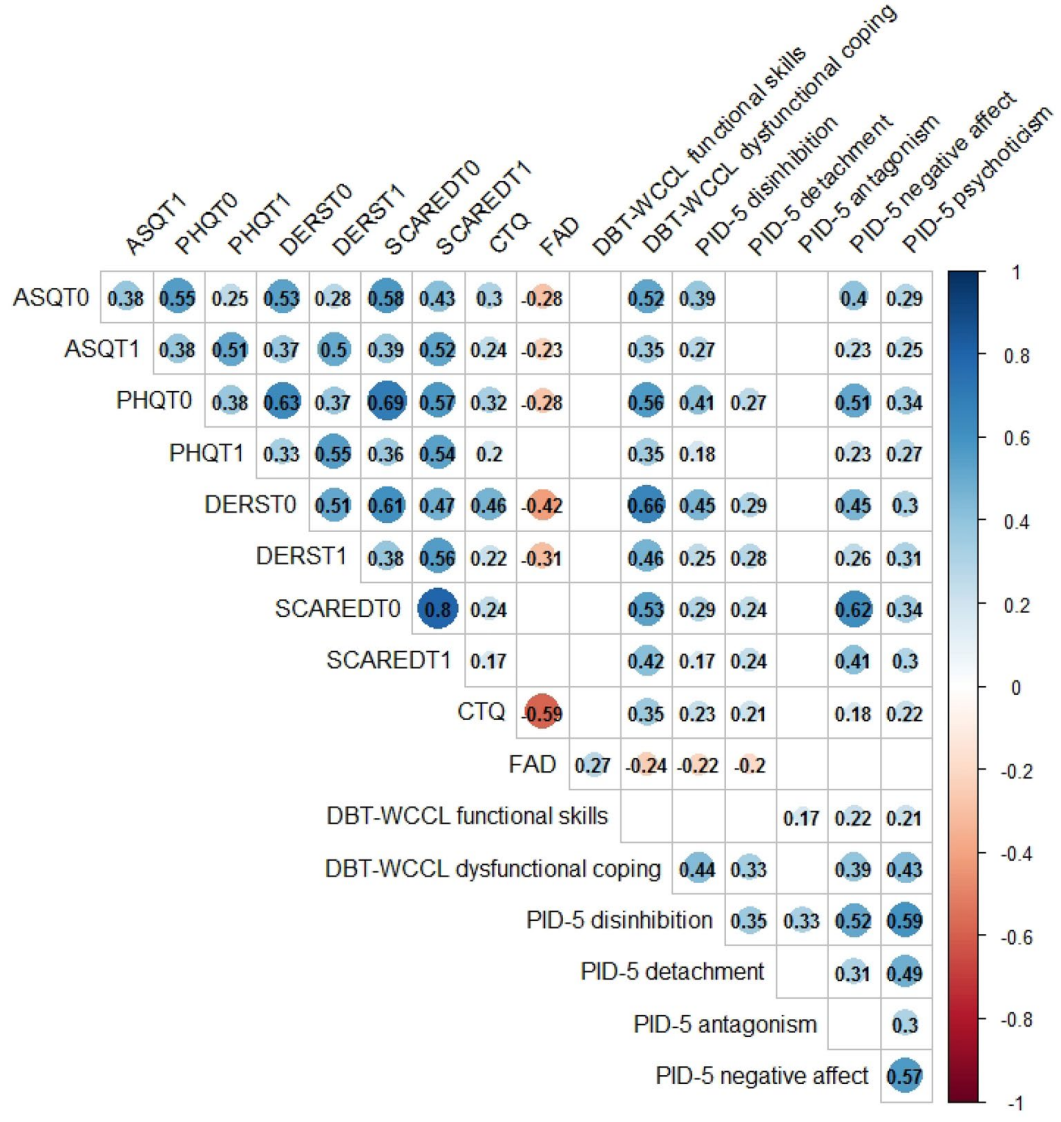
Associations between mental health indexes and risk or resilience factors: significant Spearman’s rho correlation values

Figure 3 reports the correlations between mental health indexes and potential risk or resilience factors. Both at T0 and T1, dysfunctional emotional regulation skills and childhood trauma were associated to higher levels of depression, anxiety, stress and emotional dysregulation. By contrast, better family functioning was inversely associated to all psychopathological symptoms. Among personality factors, negative affectivity and disinhibition were associated to higher scores on mental health indexes. Of note, almost all correlation coefficients are moderate, suggesting a quite robust association between variables.

### Variables associated to the category of change in mental health status over time

Participants belonging to the worsened category (found by the cluster analysis reported above) showed lower scores at baseline in stress, anxiety, depression and emotional dysregulation compared to participants belonging to the stable and improved categories. By contrast, participants belonging to the improved category showed higher scores at baseline on all mental health indexes compared to other groups (see Additional file 1: Table S2).

To quantify (through OR) the strength of the associations found by the correlation analysis, univariate multinomial logistic regression analyses were conducted. More in detail, the dependent variable was the pattern of change over time (i.e., worsened, stable, improved), while the independent variables were the change (T0–T1) of frequency in maladaptive behaviors and clinical scales (i.e., personality traits, early trauma experiences, family functioning, emotional regulation skills). The logistic regression analyses were adjusted for baseline severity of mental indexes (see Additional file 1: Figure S1).

Table 4 reports the results showing that, even when we controlled for clinical status of mental health at baseline, gender, SES and Covid-19 experience, participants who reported increased frequency of self-harm ideation (p < 0.001), self-harm behaviours (p < 0.001), binge-drinking (p = 0.005), aggressiveness (p = 0.003), and binge-eating (p = 0.003) were more likely to be worsened on mental health indexes. More in detail, students who reported a decrease in the frequency of self-harm behaviors (i.e., who had an improvement in such behavior) had more than three times higher (and 28 times) probability to be stable (improved) compared to being worsened (OR = 3.25 and OR = 28.06, p < 0.001 respectively). Students who reported a decrease in the frequency of self-harm ideation had two times (and almost seven times) higher probability to be stable (improved) compared to being worsened (OR = 2. 1, OR = 7.05; p < 0.001 respectively). Students who reported a decrease in the frequency of binge-drinking had almost four times higher probability to be stable (OR = 3.75; p = 0.005) compared to being worsened. Students who reported a decrease in the frequency of aggressiveness had two times (2.6 times) higher probability to be stable (improved) (OR 1.92, OR = 2.63; p = 0.003) compared to being worsened. Students who reported a decrease in the frequency of binge-eating had almost three times higher probability to be improved (OR 2.73; p = 0.003) compared to being worsened. Finally, none of the other clinical variables (i.e., personality traits, early trauma experiences, family functioning, and emotional regulation skills) were associated to different patterns of change in mental health over time.

**Table 4:** Results of multinomial logistic regression models assessing association between category of change in mental health status over time and risk and reliance factors

	OR (stable vs worsened [ref. category]	OR CI (stable vs worsened)	OR (improved vs worsened [ref. category])	OR CI (improved vs worsened)	p-value	Pseudo R^2^
Reduction in binge drinking^a,b^	3.75	1.30–10.81	1.34	0.51–3.54	0.005	0.23
Reduction in cannabis use^a^	1.92	0.91–4.05	1.48	0.68–3.25	0.217	0.18
Reduction in aggressiveness^a,b^	1.92	1.11–3.32	2.63	1.45–4.77	0.003	0.24
Reduction in binge eating^a,b^	1.21	0.62–2.33	2.73	1.23–6.02	0.003	0.24
Reduction in unprotected sex^a^	0.36	0.10–1.24	0.43	0.12–1.50	0.173	0.19
Reduction in self-harm behaviors^a,b^	3.25	1.42–7.42	28.06	3.23–243.38	< 0.001	0.32
Reduction in self-harm ideation^a,b^	2.10	1.10–4.01	7.05	2.44–20.37	< 0.001	0.29
CTQ	1.09	0.97–1.22	1.05	0.93–1.18	0.181	0.18
FAD	0.99	0.95–1.03	1.01	0.97–1.05	0.446	0.17
DBT-WCC skills	1.78	0.47–6.78	1.19	0.29–4.87	0.601	0.17
DBT-WCC dysf. coping	1.41	0.44–4.54	1.37	0.41–4.58	0.838	0.16
PID-5 negative affect	2.61	0.95–7.18	1.24	0.43–3.58	0.059	0.20
PID-5 detachment	0.85	0.27–2.64	0.39	0.11–1.44	0.214	0.20
PID-5 antagonism	1.25	0.40–3.89	1.01	0.30–3.39	0.846	0.17
PID-5 disinhibition	3.41	0.81–14.43	2.31	0.52–10.37	0.215	0.19
PID-5 psychoticism	0.61	0.22–1.75	0.48	0.15–1.48	0.437	0.18

The models assess the association between student groups (worsened, stable, improved as dependent variables) and potential risk or resilience factors (independent variables); adjusting for the following covariates: status of mental health at baseline, gender, SES and Covid-19 experience

Ref. category gender = males; ref. category SES = high SES; ref. category Covid-19 experience = having experienced hospitalization/death of a family member

Stable categories include values between [− 11, 11] for ASQ; between [− 7, 7] for SCARED; between [− 3, 3] for PHQ and between [− 14, 14] for DERS

*CTQ* childhood trauma questionnaire; *FAD* family assessment device; *DBTWCC* DBT-ways of coping checklist; *PID-5* personality inventory for DSM-5

^a^Change of frequency in maladaptive behaviour

^b^The adjustment for status of mental health at baseline [two categories: good, poor (ref. category)] was significant

## Discussion

The current study assessed mental health status before the Covid-19 pandemic and after one year in a sample of adolescent students living in Brescia, that along with another city in north Italy, Bergamo, were the epicenters of the first wave of Covid-19 in Italy. Participants referred a death rate from Covid-19 in line with the national estimate (13%), thus confirming that the present sample was particularly exposed to Covid-19 with a higher mortality rate compared to those reported in other European countries (Situation Report—86) [31]. The students of the present sample had been subjected to a prolonged period of restrictive measures put in place to halt the spreading of the Covid-19 pandemic. Furthermore, all the participants attended online learning for 13 months out of an 18-month school period.

In line with previous studies conducted on adolescents [6–9, 12, 32], we found an overall increase in anxiety levels and stress for future uncertainty. More in detail, we observed that specific components of anxiety—that are generalized anxiety and school anxiety—showed an ascending trend from baseline to follow-up. It has to be considered that at the follow-up assessment the participants had been back in school in presence for a few weeks after a long period of online learning and had not yet received any type of vaccination, which could represent a further stress factor. By contrast, other domains of anxiety such as panic symptoms, social anxiety, and separation anxiety remained stable. It is possible that school closures and lockdown implied a reduction of potential triggering situations such as social contacts, social pressure, bullying or other potential stressful situations. In line with this interpretation, it has to be noted that adolescents reported lower levels of perceived stress related to romantic relationships and peer pressure compared to baseline. The present data suggests the need to careful monitoring since friendships and romantic relationships constitute crucial factors during adolescence, assuring social support, independence from parents, and identity exploration [33]. Lack of social contacts could cause failure to fulfill basic human needs to belong [34]. In addition, long-term longitudinal studies demonstrated that social problems in early adolescence were associated with depressive symptoms in late adolescence and early adulthood [35].

In contrast with other studies showing an increase of depressive symptoms [9, 12, 32], no differences in the overall mean scores for depressive symptoms at follow-up emerged, neither in the proportion of youths with scores above cut-off. However, we found that half of those with scores above cut-off at baseline were still above the cut-off 1 year later, while one third of those with scores below cut-off at baseline had an increased total score and were above cut-off one year later. This latter result seems to suggest that the pandemic may have had different outcomes on youths, and this may contribute to explain why available studies show contrasting results regarding adolescents’ mental health after the pandemic [5].

Interestingly, we found that the rate of specific maladaptive behaviors (unprotected sex, self-harm ideation, self-harm behaviors, binge eating episodes, and aggressiveness) increased from baseline to follow-up. It is well acknowledged that this type of behavior often represents an attempt to manage emotional activation and to reduce emotional distress [36, 37]. In this sense, the increase in frequency of maladaptive behaviors observed in our study may represent a response to the greater psychological discomfort experienced by the students. A recent study showed that limited access to emotion regulation strategies in adolescents emerged as a significant risk factor for increased psychological stress after the pandemic [8]. Similarly, university students expressing the need for psychological support during pandemic were more likely to show dysfunctional strategies such as substance use, denial, and self-blame [38]. A large body of research shows that effective stress coping might buffer the impact of stressful events on physical and mental health [39].

Regarding the second aim of the study, we hypothesized that the Covid-19 pandemic could have had different impacts on adolescents. Results support this hypothesis, indeed we were able to distinguish the participants according to different trajectories on their mental health over time: specifically, there was a small proportion of participants who showed a worsening (15%), while others reported an improvement (34%), or a stable condition (46%) in all the psychological domains considered simultaneously (i.e., depression, anxiety, stress, and emotional dysregulation). Interesting results have emerged regarding mental health status before pandemic and trajectories of change. Indeed, youths belonging to the worsened group exhibited lower levels of psychopathological symptoms before pandemic compared to other groups. This suggests that the pandemic and restrictive measures had a negative impact on this group of adolescents, as they moved from exhibiting absent or mild symptoms to an increased level of psychopathology. This result is in line with a previous study on 252 adolescents: one third of them reported an increase in psychological stress from baseline to the first week of lockdown and they had significantly lower stress values before the pandemic compared to adolescents who did not experience increased stress [8]. We observed an opposite trend in adolescents with higher scores prior to the pandemic, indeed, they were likely to report an improvement after the pandemic. This result is in line with a previous study showing significant reduction in mental health problems for youths who had reported higher severity of symptoms before the pandemic [10]. Overall, these results reinforce our hypothesis that the pandemic and the restrictive measures may have represented a sort of protective condition for some groups of adolescents, probably by lowering exposure to triggering situations. We could speculate that the pandemic and the restrictive measures may have normalized some behaviors, such as social avoidance, social withdrawal with the consequences that social differences tended to even out. Of course, further studies with long term follow-ups to monitor the course of symptoms across time and to verify these hypotheses are needed.

The third aim of the present study was focused on the analysis of the associations between mental health indexes and risk and resilience factors. Of note, although childhood trauma, personality, and emotional regulation skills were associated to mental health status in cross-sectional evaluations, there was no association with the change in mental health after the Covid-19 pandemic. Indeed, maladaptive behaviors resulted as the only variables associated to the change in mental health status. Specifically, we found that adolescents who reported an increase in the frequency of self-harm ideation, self-harm behaviors, binge-eating, binge-drinking were more likely to be worsened compared to those who showed a decrease in the frequency of such behaviors. Of course, we cannot establish the exact direction of the association: it is possible that the worsening of psychopathological status leads to higher frequency of maladaptive behaviors. Alternatively, the increased frequency of maladaptive behaviors could have caused the worsening of psychological symptoms. Based on the present findings, it seems that the contingent reactions to pandemic (i.e. maladaptive behaviors) could be more influent than distal factors, such as early adverse experiences, family functioning, and personality in determining the trajectories over time. Overall, this seems to confirm the crucial role of emotional regulation skills in order to assure psychological wellbeing, especially during challenging periods of time, such as a pandemic and restrictive measures or as social distancing.

The study has several strengths. Firstly, this is one of the few studies reporting prospective data on mental health of adolescents during pandemic. To our knowledge, it is the first reporting data on a 1-year follow-up after the beginning of the Covid-19 pandemic, indeed available longitudinal studies have shorter follow-ups or they conducted assessments only during the first wave of the Covid-19 pandemic. Secondly, the sample has been recruited from a homogeneous geographical area, highly affected by Covid-19, and all the participants underwent the same restrictive measures for a long period of time. Thirdly, participants underwent a comprehensive assessment through standardized instruments, and this represents a main point of strength compared to previous studies. Moreover, we did not find any studies reporting data regarding maladaptive behaviors that have high clinical relevance and could be a target for possible preventive interventions. Of course, some limitations of the present study should be considered. First of all, the present sample is a convenience and self-selected sample, it cannot be representative of the entire student population located in the city of Brescia. The sample size is smaller than other studies, however it is sufficient to perform statistical analyses on different subgroups. Moreover, most of the participants were female and the recruitment was not randomized, however this unbalancing was adjusted methodologically.

## Conclusions

The present results suggests that the Covid-19 pandemic seems to have had a different impact on subgroups of student in terms of mental health. Indeed, we found a global worsening of psychological well-being only in a subgroup of adolescents, otherwise other students remained stable or improved. Of note, adolescents with the highest scores before the pandemic were likely to report an improvement after the pandemic, and vice versa. Childhood trauma, family functioning, personality traits, and emotional regulation skills were associated to mental health symptoms, however the trajectory of change across time in psychological symptoms was independent from these variables. Indeed, maladaptive behaviors (i.e., binge eating, self-harm, binge-drinking) resulted as the only variable associated to change in mental health status. Considering that maladaptive behaviors often represent strategies to manage negative emotional states, our results seem to support the usefulness of interventions aimed at strengthening adaptive emotional regulation strategies. Moreover, the overall results highlight the importance of future careful monitoring of mental health conditions and social wellbeing in this population.

## Supplementary Information

**Additional file 1: Table S1.** Descriptive statistics of the mental health index changes in the three groups found by K-means cluter analysis. **Table S2.** Mean score before pandemic by categories of change (Worsened, Stable, Improved) found by K-means cluster analysis. **Figure S1.** Dendrogram of the Hierarchical Cluster analysis (Ward’s method) on the four mental health indexes before pandemia (T0).

## Data Availability

The dataset analysed for the current study is available in Zenodo repository (https://zenodo.org/record/6327220#.YiNgLnrMJPY) (doi. https://doi.org/10.5281/zenodo.6327220).

## Abbreviations

Covid-19: Coronavirus 19
T0: Baseline assessment (November 2019–January 2020)
T1: Follow-up assessment (April–May 2021)
PHQ-9: Patient health questionnaire-9
SCARED: Screen for child anxiety related emotional disorders
ASQ: Adolescent stress questionnaire
DERS: Difficulties in emotion regulation scale
CIB: Checklist for impulsive behaviors
CTQ: Childhood trauma questionnaire
FAD: Family assessment device
DBT-WCCL: DBT-ways of coping checklist
PID-5: Personality inventory for DSM-5
MCA: Multiple correspondence analysis
OR: Odds-ratio
