# Supplementary material for: Adolescents’ mental health and maladaptive behaviors before the Covid-19 pandemic and 1-year after: analysis of trajectories over time and associated factors
Source: Child Adolesc Psychiatry Ment Health. 2022 Jun 10;16:42. doi: 10.1186/s13034-022-00474-x (PMC9186010; doi:10.1186/s13034-022-00474-x)
Supplement: Supplementary file 1 — Additional file 1: Table S1. Descriptive statistics of the mental health index changes in the three groups found by K-means cluter analysis. Table S2. Mean score before pandemic by categories of change (Worsened, Stable, Improved) found by K-means cluster analysis. Figure S1. Dendrogram of the Hierarchical Cluster analysis (Ward’s method) on the four mental health indexes before pandemia (T0). [file 13034_2022_474_MOESM1_ESM.docx]

**ADDITIONAL MATERIALS**

**Table S1. Descriptive statistics of the mental health index changes in the three groups found by K-means cluter analysis**

| **Cluster** | **Cluster size** | **Change ASQ**  **[Median] Mean** | **Change SCARED**  **[Median] Mean** | **Change PHQ**  **[Median] Mean** | **Change DERS**  **[Median] Mean** |
| --- | --- | --- | --- | --- | --- |
| 1 (Red cluster: worsened) | 23 | [-9] -8 | [-9] -9.7 | [ -4 ] -4.5 | [ -37 ] -37.9 |
| 2 (Green cluster: stable) | 75 | [-1] -2.3 | [-2] -3 | [ -1 ] -1.2 | [ -5 ] -3.4 |
| 3 (Blue cluster: improved) | 55 | [24] 24.5 | [ 3] 2.5 | [ 1 ] 2.1 | [ 12 ] 14.3 |

**Table S2. Mean score before pandemic by categories of change (Worsened, Stable, Improved) found by K-means cluster analysis**

|  | N = 23 | N = 75 | N = 55 |  |
| --- | --- | --- | --- | --- |
|  | Worsened  Mean (SD) | Stable  Mean (SD) | Improved  Mean (SD) | p-value ^£^ |
| **ASQ T0** | 67.4 (16) | 70.6 (17.7) | 87 (16.1) | **<0.001** |
| **SCARED T0** | 61.7 (12) | 63.7 (12.4) | 68.7 (11.7) | **0.024** |
| **PHQ T0** | 5.8 (3.3) | 7.5 (4.7) | 9.1 (5) | **0.014** |
| **DERS T0** | 71.2 (14.6) | 85.8 (20) | 97.9 (22.8) | **<0.001** |

£ p-value of ANOVA test

Stable categories include values between [-11, 11] for ASQ; between [-7, 7] for SCARED; between [-3, 3] for PHQ and between [-14, 14] for DERS.

**Figure S1.**  Dendrogram of the Hierarchical Cluster analysis (Ward’s method) on the four mental health indexes before pandemia (T0).


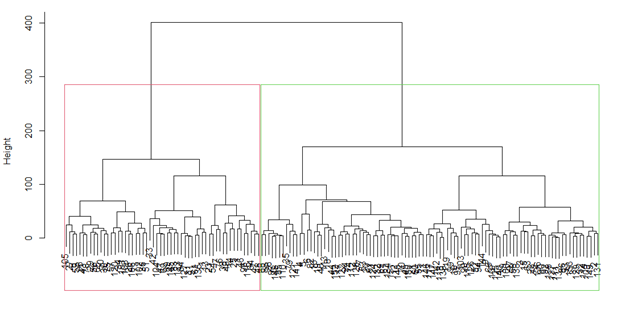


Two clusters were found: in the left red cluster belong students with a worse “clinical status of mental health” at T0 and in the right green cluster there are students with a better “clinical status of mental health”. Here below the means and standard deviation of the 4 clinical scales at T0 in the two clusters.

| **Cluster label** | **Cluster size** | **ASQ T0 ***  **Mean (SD)** | **SCARED T0 ***  **Mean (SD)** | **PHQ T0 ***  **Mean (SD)** | **DERS T0 ***  **Mean (SD)** |
| --- | --- | --- | --- | --- | --- |
| 1 (Red cluster: worse clinical status) | 55 | 92.5 (14.4) | 75.8 (9.0) | 11.5 (4.6) | 111.9 (15.8) |
| 2 (Green cluster: better clinical status) | 98 | 66.8 (13.9) | 59.2 (9.7) | 5.8 (3.3) | 74.5 (12.8) |

* Statistical differences between cluster red and cluster green: p<0.01 for all scales.

The clinical status of mental health at baseline (T0) was introduced as categorical variables [*1 vs 2* as cluster labels] in the multinomial logistic regression models showed in Table 5.
